# Urinary Retention in a Female With Subacute Combined Spinal Cord Degeneration and Vaginal Infibulation

**DOI:** 10.7759/cureus.34459

**Published:** 2023-01-31

**Authors:** Emmanuel C Okpii, Fatima Adamu-Biu

**Affiliations:** 1 Urology, North Bristol NHS Trust, Southmead Hospital, Bristol, GBR; 2 Urology, North West Anglia NHS Foundation Trust, Peterborough, GBR

**Keywords:** supra-pubic catheter, subacute combined spinal-cord degeneration, nitrous oxide abuse, vaginal infibulation, female genital mutilation, urinary retention

## Abstract

Urinary retention is a common urological condition that is more prevalent in men. It is characterized by the inability to urinate and has numerous causes. This case report describes a 29-year-old female who was admitted with a history of nitrous oxide (NO) abuse and was diagnosed with subacute combined spinal cord degeneration (SACD). The patient was found to have female genital mutilation (FGM; infibulation), which was complicated by acute urinary retention. After unsuccessful urethral catheterization, a supra-pubic catheter was inserted with no post-operative complication. The patient is currently awaiting further discussion and recommendations from a multidisciplinary team for definitive care.

## Introduction

The National Institute for Health and Care Excellence (NICE) defined urinary retention as the inability to urinate voluntarily [1]. It has various causes, including urethral obstruction, medications (such as the use of anti-muscarinic drugs, sympathomimetics, and tricyclic antidepressants), conditions that impede detrusor contractions or urethral relaxation, neurogenic causes, and postpartum or postoperative conditions [1]. Retention can be acute, chronic, or acute-on-chronic, with slight differences in its cause between males and females due to their distinct anatomy. However, the underlying physiologic mechanisms, from the sensory perception of the need to urinate to the voluntary release of urine, are similar for both sexes. Consequently, any interruption to the neural or structural pathways can result in urinary retention. Urinary retention is uncommon in women, with an estimated 3-7 cases per 100,000 women per year and a female-to-male ratio of 1:13 [2]. This report highlights a complex case of urinary retention in a female with subacute combined spinal cord degeneration (SACD) and female genital mutilation (FGM).

## Case presentation

A 29-year-old female was brought to the ED by ambulance, complaining of a tingling sensation in her hands and weakness in her lower limbs. Her sister had called for medical assistance after discovering that the patient had been inhaling nitrous oxide (NO) with the goal of feeling euphoric. The patient had no intention of self-harm even though she previously experienced a psychotic episode 11 months ago. She also reported using cannabis recreationally. On examination, the patient had a Glasgow Coma Scale (GCS) score of 15/15 and normal cranial nerve function. However, neurological examination revealed normal tone and power in her upper limbs with altered sensation in both hands. Power was 4/5 in her lower limbs bilaterally, with absent ankle reflexes and decreased proprioception. Gastrointestinal examination revealed glossitis. Other examinations, including respiratory and cardiovascular, were normal. The patient was taking omeprazole, sertraline, and quetiapine but had been noncompliant with her medication regimen. While on the ward, she reported not urinating since the previous evening, and an abdominal examination revealed a soft abdomen with tenderness over the supra-pubic area. A bladder scan showed 750 ml of urine in her bladder. Based on her clinical history and examination, a differential diagnosis of metabolic myeloneuropathy secondary to vitamin B12 deficiency was suspected.

Investigation

The patient's laboratory results (Table 1) revealed low vitamin B12, normal hemoglobin with elevated mean corpuscular volume, and macrocytosis on the blood film. Renal function, thyroid-stimulating hormone (TSH), and copper (Cu) were normal. The intrinsic factor antibody was positive.

**Table 1:** Laboratory results.

Laboratory Test	Result	Normal Range
Sodium (Na)	142	135-145 mmol/L
Potassium (K)	3.6	3.5-5.5 mmol/L
Urea (Ur)	3.6	2.5-7.8 mmol/L
Creatinine	52	45-84 umol/L
Estimated Glomerular Filtration Rate (eGFR)	>90	>90 ml/min
Thyroid-Stimulating Hormone (TSH)	2.71	0.38-5.33 mIU/L
Copper (Cu)	12.9	11-25 umol/L
Vitamin B12	130	180-900 ng/L
Haemoglobin (HB)	136	120-150 g/L
Mean Corpuscular Volume (MCV)	110.2	83-100 fL
Blood film	Macrocytosis	Normocytosis
Intrinsic Factor Antibody	Positive	Negative

**Figure 1:**
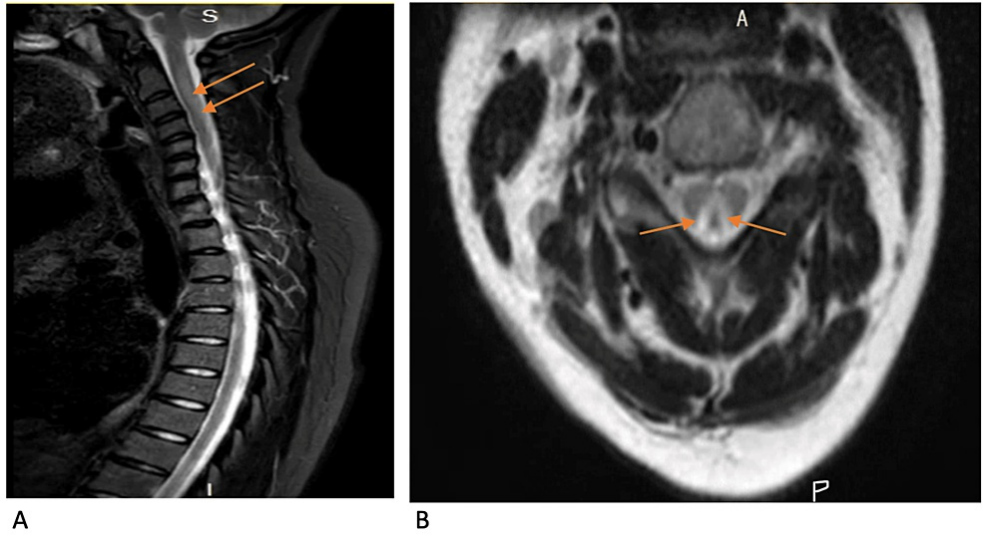
MRI spine of the index patient. (A) Sagittal section showing T2 hyperintensity in the dorsal cervical spinal cord indicated by orange arrows. (B) Axial section showing T2 hyperintensity with inverted V sign in cervical spinal cord indicated by orange arrows.

An MRI of her spine revealed a long segment of abnormal T2 hyperintensity in the dorsal cord extending from the C3 to C5/6 levels in a triangular shape (Figure 1). The spinal canal was normal, with no compression of the spinal cord. Normal conus was located at L1/2, and cauda equina was also normal. The marrow signal was also normal. These findings were consistent with SACD. An MRI of her head was normal.

Treatment

**Figure 2:**
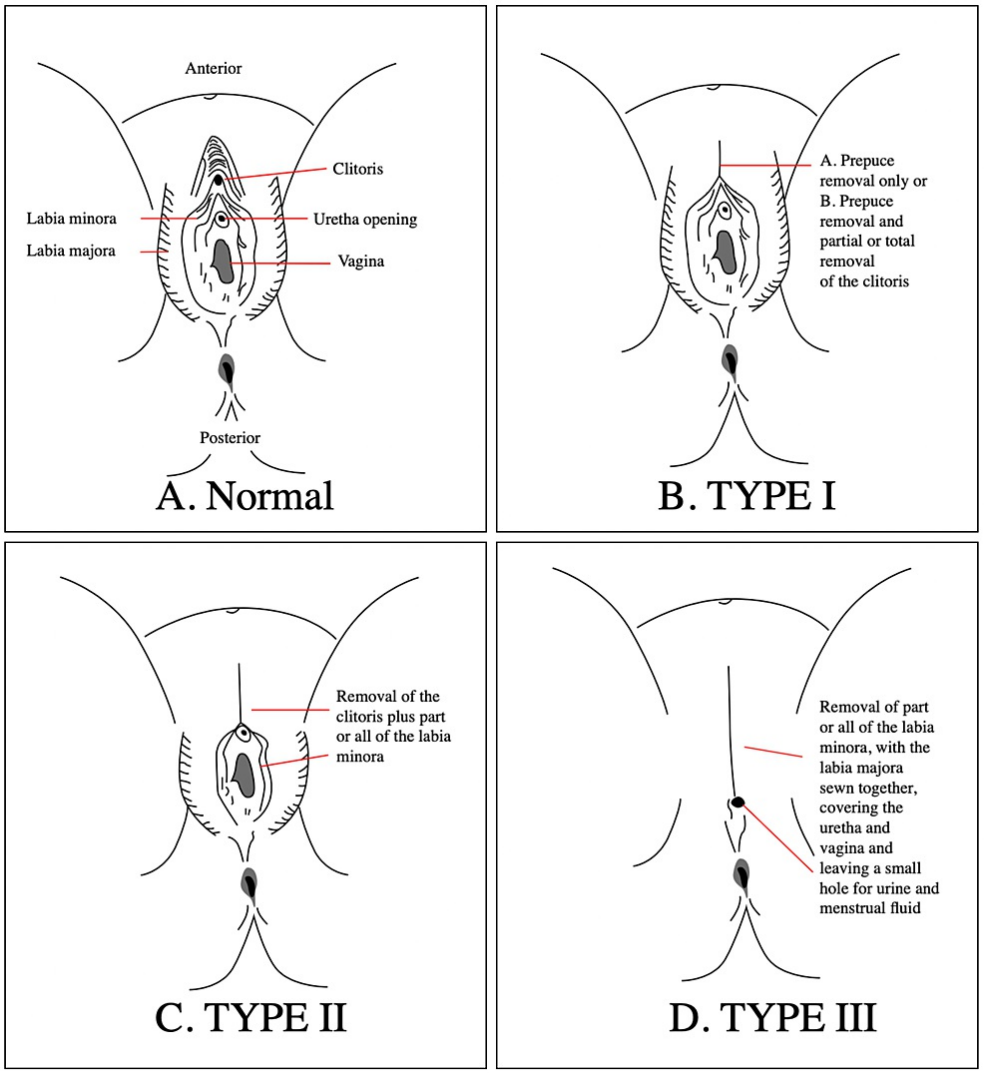
Diagrammatic representation of female genital mutilation. Source: Wikipedia.

The patient's history of NO abuse, examination findings of altered neurology, urinary retention, laboratory results, and imaging led to a diagnosis of SACD secondary to vitamin B12 deficiency. She was started on vitamin B12 injections, but multiple attempts at urethral catheterization were unsuccessful. This prompted a referral to the on-call urology team, who, upon examination, found the patient to be in mild discomfort from a full bladder. Further examination of the urogenital system revealed type 3 female genital mutilation (infibulation) (Figure 2). Attempts at urethral catheterization with a guide wire were unsuccessful, and the patient was counselled on the need for a supra-pubic catheter insertion under local anaesthesia.

**Figure 3:**
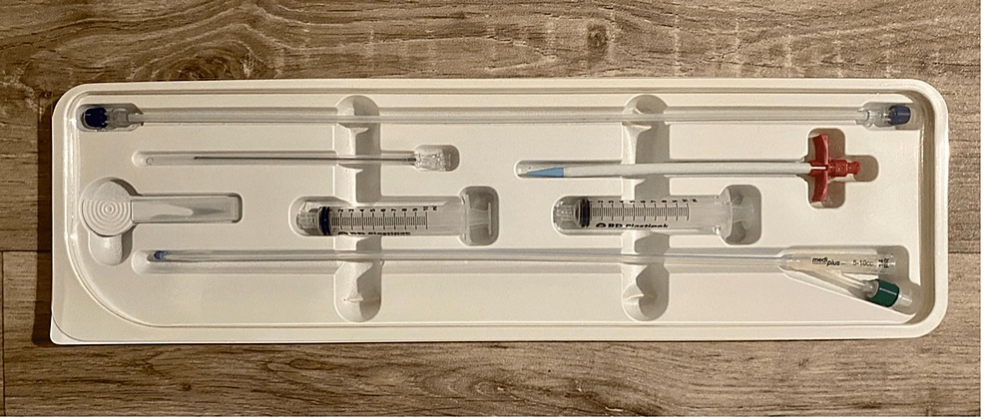
MEDIPLUS supra-pubic catheter kit.

The patient consented to the procedure, and the area was cleaned and draped. Local anaesthesia was applied and using the Seldinger technique with a MEDIPLUS kit (Figure 3), a 14Ch 2-way silicone catheter was inserted under ultrasound guidance and retained with 10 ml of sterile fluid. Post-insertion, the catheter drained 950 ml of clear urine, providing immediate relief to the patient. She is awaiting discussion by a multidisciplinary team for definitive management.

## Discussion

SACD is a neurological complication of vitamin B12 deficiency. It is characterized by degeneration of the dorsal and lateral columns of the spinal cord due to demyelination [3]. There are multiple causes of vitamin B12 deficiency, including nutritional deficiency, gastric abnormalities (such as gastric surgery or gastritis), small bowel disease, pancreatic disease, drug-induced (such as the use of gastric acid suppressants, metformin or NO), fish tapeworm infestation, and genetic abnormalities. The patient had a history of NO abuse, low levels of vitamin B12, anti-intrinsic factor antibody, and imaging confirming her diagnosis of SACD. Micturition abnormalities have been reported in patients with SACD but have not been exhaustively and systematically evaluated. However, a prospective study by Misra UK et al. has described both voiding and storage micturition disturbances in patients with SACD [2-4]. Unfortunately, our patient was also a victim of childhood genital mutilation. FGM is the partial or total removal of the female external genitalia or other deliberate injuries to the female genital organs for cultural or non-therapeutic reasons [5]. The WHO estimates that over 200 million girls and women alive today have undergone FGM in 30 countries in Africa, the Middle East, and Asia, where it is practiced. Although FGM is not a recorded practice in the UK, transcontinental migration has made it possible for UK healthcare professionals to encounter such patients.

FGM is classified into four major types (Table 2) [5].

**Table 2:** WHO classification of female genital mutilation.

Types	Description
Type 1	Partial or total removal of the clitoral glans and/or the prepuce/clitoral hood
Type 2	Partial or total removal of the clitoral glans and the labia minora, with or without removal of the labia majora
Type 3 (Infibulation)	Narrowing of the vaginal opening through the creation of a covering seal, formed by cutting and repositioning the labia minora or labia majora, sometimes through stitching, with or without removal of the clitoral prepuce/clitoral hood and glans
Type 4	Other harmful procedures to the female genitalia for non-medical purposes (e.g. pricking, piercing, incising, scraping and cauterizing the genital area)

FGM may result in immediate complications, such as severe pain and bleeding; long-term complications, such as psychological and psychosexual trauma, infertility, and susceptibility to bacterial vaginosis; and obstetric complications, including perinatal death [6]. The patient was found to have type 3 FGM and reports passing urine from an opening in the infibulated genital (Figure 2). Her urethral catheterization was unsuccessful due to difficult access to the urethral opening. The blind probe of the small vulval opening using a sensor guide wire and s-shaped dilators failed to gain access into the urethra. Due to the patient's discomfort and soreness of the perineum, she consented to undergo the insertion of a supra-pubic catheter under local anaesthesia.

Outcome

The patient is currently under the care of the neurology team, where she is receiving vitamin B12 treatment for her SACD. For the first time, she has permitted the disclosure of her FGM status to healthcare professionals and has been referred to an FGM clinic upon discharge. Urologically, her catheter is draining clear urine, and she is awaiting further discussion by the multidisciplinary team. She will undergo a change of supra-pubic catheter 10 weeks from the date of insertion.

## Conclusions

Urinary retention, when acute, can present as a urological emergency. However, it is easily treated if identified early and the appropriate tools are available. In some cases, however, patients may present with complex urinary retention that requires a multidisciplinary team approach to its management, as exemplified by our index case.

## References

[1] (2023). NICE: Urinary retention: treatment summaries. https://bnf.nice.org.uk/treatment-summaries/urinary-retention/.

[2] Misra UK, Kalita J, Kumar G, Kapoor R (2008). Bladder dysfunction in subacute combined degeneration: a clinical, MRI and urodynamic study. J Neurol.

[3] Qudsiya Z, De Jesus O (2022). Subacute Combined Degeneration of the Spinal Cord. https://www.ncbi.nlm.nih.gov/books/NBK559316/.

[4] Leslie SW, Rawla P, Dougherty JM (2022). Female Urinary Retention. https://www.ncbi.nlm.nih.gov/books/NBK538497/.

[5] (2023). World Health Organization. Female genital mutilation. https://www.who.int/news-room/fact-sheets/detail/female-genital-mutilation.

[6] Okwudili OA, Chukwudi OR (2012). Urinary and genital tract obstruction as a complication of female genital mutilation: case report and literature review. J Surg Tech Case Rep.

